# L-type calcium channel blockers, morphine and pain: Newer insights

**DOI:** 10.4103/0019-5049.63652

**Published:** 2010

**Authors:** Rakesh Kumar, RD Mehra, S Basu Ray

**Affiliations:** Department of Anatomy, All India Institute of Medical Sciences, New Delhi - 110 029, India

**Keywords:** Formalin test, morphine, naloxone reversal test, nimodipine, pain, rat, synergism

## Abstract

Earlier, we had reported that co-administration of opioids and L-type calcium channel blockers (L-CCBs) like diltiazem could prove useful in the treatment of cancer pain. Much of this report was based upon earlier published work involving animal models of pain exposed to brief periods of noxious radiant heat without any tissue injury. However, pain in clinical situations usually result from tissue injury. Thus, the aim of the current investigation was to study the analgesic effect of this combination of drugs in the rat formalin test which is associated with actual tissue injury. Wistar rats (n=60) received either L-CCB (nifedipine/nimodipine/verapamil/diltiazem i.p.) or morphine (s.c.) or both drugs. The formalin test was done 30 min after morphine or placebo injection. The naloxone reversal test was also done. Administration of L-CCBs alone, particularly diltiazem, increased pain in the formalin test. In contrast, co-administration of these L-CCBs with morphine led to decreased pain response, though statistically significant decrease was noted only with nimodipine + morphine. Naloxone reversed this analgesic effect, indicating that it was primarily an opioid-mediated effect. The results show that administration of L-CCBs alone may prove counterproductive in the therapeutic management of pain (anti-analgesic effect). However, co-administration of both drugs (morphine and nimodipine) in quick succession could lead to adequate pain relief.

## INTRODUCTION

Several studies on the antinociceptive effect of L-type calcium channel blockers (L-CCBs) + opioids like morphine have reported significantly higher antinociceptive effect than that produced by either of the drugs administered alone.[1‐3] These studies were conducted in animals after co-administration of the drugs through the *systemic* route. In many cases, L-CCBs did not have any antinociceptive effect by itself, suggesting a synergistic interaction.[1‐3] However, these experimental works depended upon exposing the animals to brief thermal stimuli (phasic pain model) as in the tail-flick and the hot plate tests. Pain models based upon occurrence of continuous pain as in the formalin test (tonic pain) have not been used except in one study, which was conducted in mice.[4] It was observed in this study that administration of diltiazem (20,40,80 mg/kg), verapamil (10,20 mg/kg), flunarizine (20,40,80 mg/kg) or nimodipine (20,40,80 mg/kg) did not produce any analgesic effect in the tail-flick test. However, in the formalin test, all except verapamil significantly reduced the pain response. Furthermore, co-administration of L-CCBs and morphine (5mg/kg) led to increased antinociceptive response, both in the tail-flick and the formalin tests in both the early (0-5 min) and late (25-30 min) phases. In contrast, with the dosage (morphine/nimodipine/nifedipine – 2 mg/kg; verapamil – 5 mg/kg; diltiazem – 10 mg/kg) used in the present study, no statistically significant antinociception was observed in comparison to physiological saline (placebo) in the formalin test. It was hypothesized that a significantly higher analgesic effect, if any, after co-administration of both groups of drugs, would indicate a synergistic interaction.

Regarding the analgesic effect of L-CCBs after *intrathecal* administration, it has been reported that administration of either nifedipine or verapamil decreased pain response in the late phase of the formalin test without affecting the early phase.[5] Conversely, BAY K-8644 (antagonist of L-CCBs) increased nociceptive response in the late phase. However, a different study noted that diltiazem and verapamil but not nifedipine or nimodipine produced significant antinociception in the late phase only.[6] Thus, it has been suggested that spinal L-VSCCs could play a moderate role in the generation of pain response.[7] Previous studies in our laboratory have also shown synergistic interaction between nimodipine and morphine after intrathecal administration in the tail-flick test.[8]

The efficacy of L-CCBs + morphine co-administration *in humans* has also been studied, particularly with reference to postoperative pain. Recently, it was reported that administration of nimodipine + morphine led to higher morphine consumption (anti-analgesic effect).[9] Similarly, Zarauza *et al*. too did not observe any opioid-sparing effect of L-CCB during postoperative pain.[10] However, Carta *et al*. 1990 had noted higher analgesic effect after co-administration of nifedipine and morphine.[11]

The formalin test depends upon direct activation of nociceptors in the early phase (0-5 min) and inflammation-related activation of nociceptors and of spinal neurons in the late phase (15-60 min).[12] Thus, the formalin test may simulate pain in clinical conditions, which is usually caused by injury and/or inflammation.

## METHODS

### Animals

Experiments were performed on adult male Wistar rats (weighing 200-250 gm). These animals were procured from the Central Animal Facility, All India Institute of Medical Sciences and housed under standard conditions with a 12h/12 h day/night cycle and access to food and water *ad libitum*. The permission for experiment on animals was taken from Institutional Animals Ethics Committee at A.I.I.M.S. Rats were acclimatized for 1 h in a glass observation chamber (30 cm diameter, 20 cm high) before drug administration.

### Drugs

Morphine sulphate (15 mg/ml ampoules) was obtained from a Government pharmacy after obtaining permission from the Narcotics Commissioner. Nimodipine and Nifedipine were dissolved in a vehicle, consisting of polyethylene glycol: physiological saline (0.9% NaCl): ethanol in 2:2:1 ratio under dim light as standardized previously.[13] Diltiazem and verapamil were dissolved in Water for injection I.P. All the L-CCBs were from Sigma (U.S.A.). Stock solution of 10% formalin (Qualigens, India) was prepared in sodium phosphate buffer.[14] It was diluted with normal saline to make 5% formalin solution at the time of the experiment.

### Experimental procedure

Animals (n=60) were randomly divided into groups and administered the following drugs: Group I – Physiological saline, Group II – Morphine (2 mg/kg), Group III – Nifedipine (2 mg/kg), Group IV – Nimodipine (2 mg/kg), Group V – Verapamil (5 mg/kg), Group VI – Diltiazem (10 mg/kg), Group VII - Nifedipine (2 mg/kg) + Morphine (2 mg/kg), Group VIII - Nimodipine (2 mg/kg) + Morphine (2 mg/kg), Group IX – Verapamil (5 mg/kg) + Morphine (2 mg/kg) and Group X – Diltiazem (10 mg/kg) + Morphine (2 mg/kg). L-CCBs were injected intraperitoneally 20 min before morphine injection through subcutaneous route and formalin was injected 30 min after morphine injection. Group I received saline twice at an interval of 20 min while Group II received saline in place of L-CCB, 20 min before morphine. Similarly, Groups III – VI received saline instead of morphine 20 min after L-CCB administration. Preliminary experiments were done to determine the individual doses of morphine and L-CCBs, which would not produce an antinociceptive effect. Also, injections of vehicle alone did not produce an antinociceptive effect (data not shown).

### Formalin test

**Figure 1:**
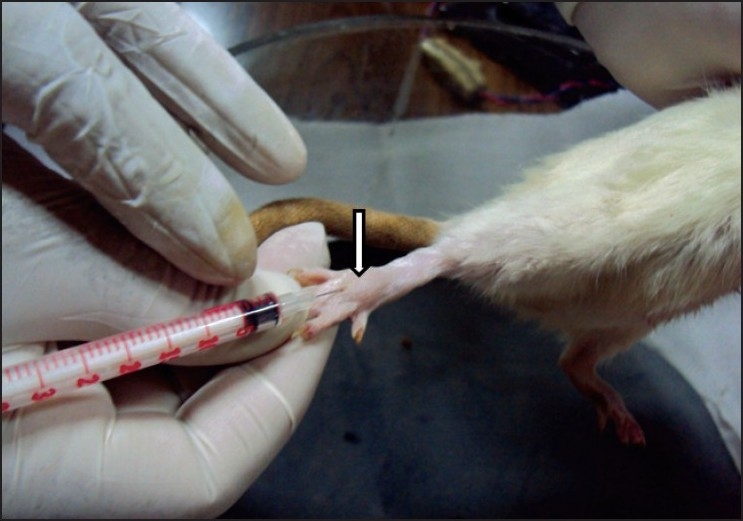
Subcutaneous administration of 5% formalin in the dorsum of the hindpaw of the rat, which has raised a small bleb (arrow).

Rats were injected 50*μ*l of 5% formalin solution subcutaneously into the dorsal surface of the right hind paw with a 30 gauge needle [Figure 1].[13] This produces a characteristic *biphasic* flinching behaviour, which can be manually counted. The intervening period between the two phases (5-15 min) is a relatively quiescent period with decreased number of flinches. Each rat was placed individually in the observation chamber and the number of flinches was counted for a period of 60 min in 5 min bins (between 0-5 and 15-60 min) by an observer, who was blinded to the drugs administered to the rats. At the end of this period, the animals were euthanized by overdose of ether inhalation. Throughout the experimental procedure, attempts were made to reduce discomfort to animals as far as possible and to keep the number of animals to the minimum.

### Naloxone reversal test

Wistar rats (n=6) were administered nimodipine and morphine as in group VIII above. Naloxone hydrochloride (5 mg/kg; Samarth Life Sciences, India) was injected intraperitoneally 30 min after morphine. Formalin test was done 10 min after naloxone administration.

### Statistical analysis

The occurence of flinches was expressed as Mean ± Standard Error of Mean. Occurrence of flinches in Phases I (0-5 min) and II (15-60 min) was determined. Group comparison was made with one-way ANOVA followed by Bonferroni multiple group comparison test (post hoc) using GraphPad Prism (GraphPad software, San Diego, CA). Student's *t*-test (unpaired) was also used for comparison between groups. A *P* value < 0.05 was considered to be statistically significant.

## RESULTS

**Figure 2:**
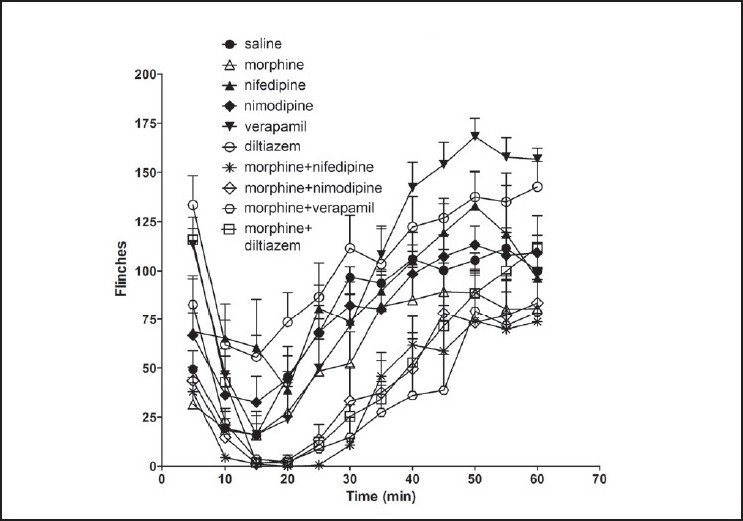
Incidence of flinching behaviour in rats after formalin injection shown in 5 min bins. In phase I (0-5 min), all the groups showed higher number of flinches than morphine. In phase II, groups receiving L-CCBs alone showed higher flinching behaviour than morphine. Groups, which had received L-CCB + morphine showed lower number of flinches. Values are shown as mean ± S.E.M.

**Figure 3:**
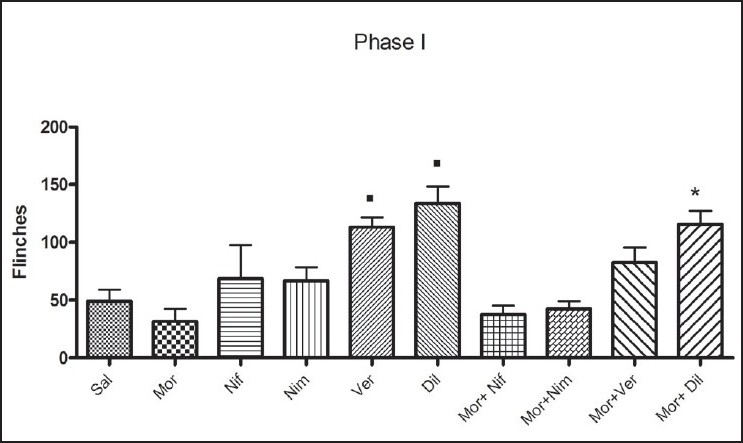
Analysis of flinching behaviour in Phase I (0-5 min): Groups of rats, which had received verapamil and diltiazem showed significantly higher number of flinches in comparison to morphine (■). Co-administration of L-CCBs + morphine did not show any beneficial effect with reference to the morphine treated group. In fact, diltiazem + morphine showed significantly higher flinches than morphine alone (*). Values are shown as mean ± S.E.M. Statistical evaluation was done by ANOVA. Significance was set at *P*<0.05.

**Figure 4:**
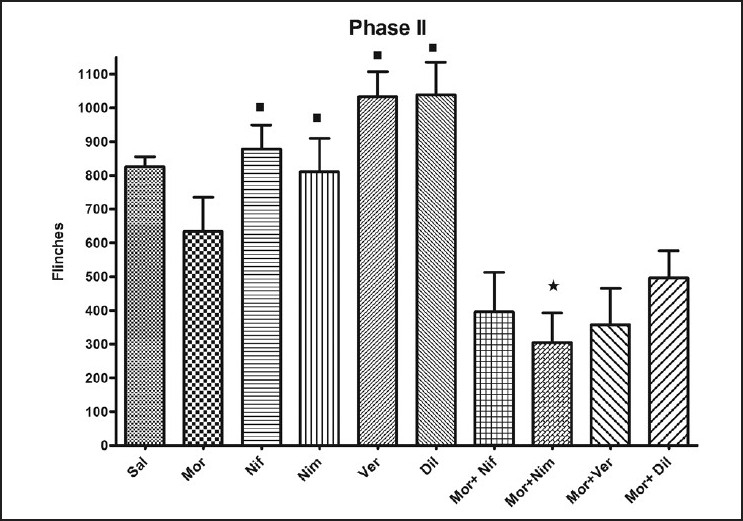
Analysis of flinching behaviour in Phase II (15-60 min): Co-administration of L-CCBs + morphine reduced the number of flinches in comparison to morphine alone. However, only nimodipine + morphine showed significant decrease of flinches with reference to the morphine treated group (*). Administration of L-CCBs alone led to significant increase in the flinching behaviour in comparison to the corresponding L-CCB + morphine treated group (■). Values are shown as mean ± S.E.M. Statistical evaluation was done by ANOVA and Student's t-test (for morphine + nimodipine group in comparison to morphine group). Significance was set at *P*<0.05.

All the groups showed biphasic formalin-induced flinching behaviour [Figure 2]. Increased number of flinches was noted for the first 5 min (Phase I) and during 15-60 min (Phase II). The morphine treated group showed decreased number of flinches during both phases though it did not show significant reduction in comparison to the saline treated group [Figures 3 and 4]. Administration of L-CCBs increased the flinching response in both phases in comparison to morphine. In phase I, this was significantly higher for diltiazem and verapamil. In phase II, though the L-CCBs did not significantly increase the flinching response in comparison to morphine, it was significantly higher than the flinches resulting from co-administration of L-CCBs + morphine. Also, increased analgesic response for L-CCBs + morphine in comparison to morphine was not uniformly observed. In phase I, diltiazem + morphine produced significantly higher flinches than morphine alone.

**Figure 5:**
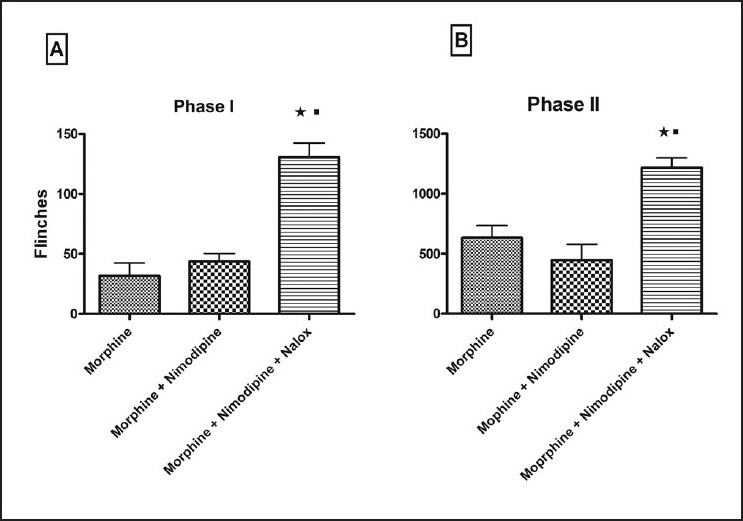
Naloxone reversibility of flinching behaviour. Naloxone significantly reversed flinching behaviour of the nimodipine + morphine treated group (*) as well as morphine treated group (■) in both phase I (A) and phase II (B). Values are shown as mean ± S.E.M. Statistical evaluation was done by ANOVA. Significance was set at *P*<0.05.

In Phase II, L-CCBs + morphine decreased flinching behaviour in comparison to morphine. When this data (Different L-CCBs + Morphine *vs*. Morphine) was analysed by ANOVA, statistically significant difference was not observed. However, Student's *t*-test revealed significant decrease of flinching behaviour in the nimodipine + morphine group with relation to morphine. Administration of naloxone reversed this inhibition of flinching behaviour, noted after morphine + nimodipine administration in both phase I and II [Figure 5].

No obvious side effect was observed after administration of the drugs.

## DISCUSSION

The present study shows that systemic administration of L-CCBs alone increased the pain response in the formalin test in rats. In phase I, verapamil and diltiazem produced significantly higher nociception than morphine alone. Even in phase II, all the L-CCBs produced significantly higher nociception than co-administration of morphine + the corresponding L-CCBs. This is contradictory to earlier studies on L-CCBs, which have noted either an absence or an increase in the antinociceptive effect, particularly at high doses.[1‐4] In fact, the present study is the first report documenting higher nociception in animals after L-CCB administration. The only other study to have investigated this effect in the formalin test had observed increased antinociception in both phases.[4] This difference could be related to the duration of the observation period in phase II, which was for 5 min only (25-30 min). Moreover, the dose of morphine was higher in the previous study. The result of the current work are also different from that of earlier studies involving intrathecal administration of L-CCBs, which had noted significantly higher antinociceptive effect in phase II of the formalin test.[5,6]

Regarding the antinociceptive effect of L-CCBs + morphine or other related opioids, majority of the earlier studies reported significantly higher antinociception in comparison to morphine/opioid alone.[1‐4,14] Similarly, in the present study, all the L-CCBs in combination with morphine produced a moderate but non-significant decrease in phase II, except nimodipine (significance was noted with Student's *t*-test). Even with morphine + nimodipine, significant decrease was absent when data was analyzed by ANOVA. This was contrary to our hypothesis. Possibly, it was due to the low dose of morphine (which did not even produce significant inhibition of flinches with reference to saline) used for the study. The low dose of morphine (2 mg/kg) was used to replicate the clinical situation where minimum doses of opioids are prescribed for avoiding side effects like respiratory depression. This effect was reversed by naloxone (5mg/kg) indicating that it was primarily mediated by opioid receptors. A recent study on adrenalectomized rats also observed that nimodipine was more effective than other L-CCBs in the tail-flick and hot plate tests.[15]

In humans, studies on the therapeutic efficacy of L-CCBs + opioids have produced conflicting results in the treatment of postoperative pain. For example, it was recently reported that nimodipine actually inhibited the analgesic effect of morphine in patients undergoing knee replacement surgery.[9] Based upon the findings of the present study, an explanation for this seemingly contradictory results may be extended. In the study of Casey *et al*. (2006), patients received 90 mg of nimodipine 1 hr before surgery.[9] The surgery continued for slightly over two hours. This time was enough for the drug to produce its effect, which would be to increase nociception. 0.5% bupivacaine (but no opioid) was used for spinal anaesthesia in these patients. On completion of surgery, the patients needed higher doses of morphine to counteract the deleterious effect of nimodipine (anti-analgesic effect). This anti-analgesic effect of nimodipine was absent in Zarauza's study because it was administered during the postoperative period along with morphine.[10] In the study by Carta *et al.,* a slow release preparation of nifedipine was used (half-life of 15.2 ± 4.3 h), which might have delayed its antianalgesic action.[11]

Though no side effects were observed in the present study, preliminary studies in our laboratory have shown that chronic administration of high doses of nimodipine (>8 mg/kg) in rats increased opioid-induced (10 mg/kg thrice daily) gastrointestinal stasis (unpublished observation). Also, higher doses of nimodipine in humans may also produce dizziness, hypotension, aggravation of myocardial ischaemia, pulmonary edema and muscle weakness.[16] Finally, in a recently reported study, it has been convincingly shown by gene knockdown procedure that L-type voltage-sensitive calcium channels do indeed play an important role in pain processing at the level of the spinal cord.[17]

In conclusion, the results show that nimodipine + morphine may be useful in the treatment of pain with the following caveats: A titration of the doses of both drugs and administration in quick succession are required to avoid their undesirable side effects. Certainly, further studies in murine models with actual postoperative/cancer pain would throw further light on this subject.
